# Salvador–Warts–Hippo pathway regulates sensory organ development via caspase-dependent nonapoptotic signaling

**DOI:** 10.1038/s41419-019-1924-3

**Published:** 2019-09-11

**Authors:** Lan-Hsin Wang, Nicholas E. Baker

**Affiliations:** 1 Graduate Institute of Life Sciences, National Defense Medical Center, 161 Sec 6, Minquan E. Rd, Taipei, 11490 Taiwan; 2 Department of Genetics, Albert Einstein College of Medicine, 1300 Morris Park Avenue, Bronx, NY 10461 USA; 3 Department of Developmental and Molecular Biology, Albert Einstein College of Medicine, 1300 Morris Park Avenue, Bronx, NY 10461 USA; 4 Department of Ophthalmology and Visual Sciences, Albert Einstein College of Medicine, 1300 Morris Park Avenue, Bronx, NY 10461 USA

**Keywords:** HIPPO signalling, Differentiation

## Abstract

The fundamental roles for the Salvador–Warts–Hippo (SWH) pathway are widely characterized in growth regulation and organ size control. However, the function of SWH pathway is less known in cell fate determination. Here we uncover a novel role of the SWH signaling pathway in determination of cell fate during neural precursor (sensory organ precursor, SOP) development. Inactivation of the SWH pathway in SOP of the wing imaginal discs affects caspase-dependent bristle patterning in an apoptosis-independent process. Such nonapoptotic functions of caspases have been implicated in inflammation, proliferation, cellular remodeling, and cell fate determination. Our data indicate an effect on the Wingless (Wg)/Wnt pathway. Previously, caspases were proposed to cleave and activate a negative regulator of Wg/Wnt signaling, Shaggy (Sgg)/GSK3β. Surprisingly, we found that a noncleavable form of Sgg encoded from the endogenous locus after CRISPR-Cas9 modification supported almost normal bristle patterning, indicating that Sgg might not be the main target of the caspase-dependent nonapoptotic process. Collectively, our results outline a new function of SWH signaling that crosstalks to caspase-dependent nonapoptotic signaling and Wg/Wnt signaling in neural precursor development, which might be implicated in neuronal pathogenesis.

## Introduction

The Salvador–Warts–Hippo (SWH) pathway has been recognized as a significant regulator for growth control, tissue regeneration, and stem cell pluripotency^1,2^. It has also been found to play important roles in cancer metastasis^3–5^. Originally identified as a prominent regulator of organ size in *Drosophila*, this pathway is highly conserved from fly to mammals. The regulation of SWH pathway depends on its response to various upstream stimuli through intercellular junctions, including adhesion cues through cell–cell contact, polarity, extracellular signal, mechanical signals, and cellular stress. Core components of the SWH pathway comprise a kinase cascade, which is the main regulation modulating SWH signaling. The Ste20 family kinase Hpo (MST1/2 in mammals) forms a heterodimer with the adapter protein Sav (SAV or WW45 in mammals), thereby promoting their interaction with the serine/threonine kinase Wts (LATS1/2 in mammals). Hpo subsequently activates Wts activity via phosphorylation^6,7^. The activated Wts kinase operates with its cofactor Mats (MOB1A/B in mammals) to phosphorylate Yorkie (Yki; YAP and TAZ in vertebrates), which is a transcriptional coactivator and serves as the final effector of the Hippo signaling. Yki/YAP/TAZ lacks DNA-binding motif but binds to the promoters of target genes through interacting with Scalloped (Sd, TEAD1-4 in mammals) or other transcription factors^6^. By activating target gene expression, Yki/YAP/TAZ plays important roles in controlling cell growth, proliferation, and survival. Wts kinase inhibits transcriptional activity of Yki/YAP/TAZ through nuclear export, cytoplasmic retention, and protein degradation^8–10^. Phosphorylation-independent regulations also exist. Yki can directly bind to Hpo, Wts and the FERM-domain containing adapter protein expanded (Ex), and sequesters Yki in the cytoplasm^11,12^. In addition to intrinsically regulates Yki activity, Ex, as an apical junctions-localized protein, also transduces signaling cues through binding to the apical membrane protein Crumbs (Crb, CRB3 in mammals)^13–16^. In addition, *ex* is a downstream target gene of Yki, thereby forming a feedback regulatory loop of Hippo pathway^17^. It has been demonstrated that activation of the SWH pathway through elevating *expanded* (*ex*) levels is required to eliminate the inappropriately differentiating neurons during development^18,19^. However, whether SWH pathway has any roles in normal neurogenesis remained unclear.

Intriguingly, hypomorphic *ex* mutants often differentiate supernumerary sensory bristles^18^. Bristles are a component of the *Drosophila* peripheral nervous system and can be divided into macro- (large bristles) and microchaetae (small bristles) according to their size and position. *Drosophila* notum is a classical model to study pattern formation because each macrochaetae develop in precise positions and microchaetae appears in a characteristic density pattern^20^. Each of these external sensory organs comprises five cells (hair, socket, neuron, sheath cell, and glial cell) that are generated through asymmetric cell divisions of single sensory organ precursor (SOP) cell^21,22^. The accuracy of bristle patterns on the adult body depends on the correct SOP cell positioning. The phenotype of *ex* mutations promoted us to study in depth how *ex* mediates sensory organ development.

Caspase activation has been implicated in SOP development through a caspase-dependent nonapoptotic machinery. This caspase-dependent machinery is thought to be required for cleavage and activation of a negative regulator of Wingless (Wg)/Wnt signaling, Shaggy (Sgg)/GSK3β, in SOP cell formation^23^. By studying how *ex* takes part in SOP development, we discovered a crosstalk between SWH pathway and caspase-dependent nonapoptotic signaling mediated through Wg pathway. Interestingly and unexpectedly, we found Sgg might not be the main target of the caspase-dependent nonapoptotic event.

## Materials and methods

### Mutants and transgenes

*ex*^*1*^, *ex*^*e1*^, *ex*^*697*^ ^24^; *Diap*^*1*^ ^25^; *Diap1*^*4*^ ^26^; *dsh*^*3*^ ^27^; *arm*^*2*^, *arm*^*3*^ ^28^ are loss-of-function or null alleles. Other transgenes used in this study include *UAS-ex-RNAi* (BDSC BL#28703); *UAS-yki*, *UAS-yki*^*S168A*^ ^10^; *UAS-dTCF[DN]* (BDSC BL#4784); *UAS-dTCF-RNAi* (BDSC BL#26743); *dpp*^*40C6*^*-Gal4*^29^; *Ex*^*Intron3*^*-GFP*^18^.

### Immunohistochemistry and histology

Preparation of wing discs for immunostaining and adult notum for light microscope was performed as described previously^30^. Confocal imaging was performed using Leica SP2, SP8 and Zeiss LSM 880 microscopy. Primary antibodies used were anti-Sens (guinea pig, a gift from H. Bellen); GFP (rat, NACALAI TESQUE# GF090R). Photograph of adult notum was carried out using Leica MZFLIII microscope and Nikon SMZ1500 microscope.

### CRISPR/Cas9-based genome editing of *sgg* gene

By using CRISPR/Cas9-mediated genome editing, mutagenesis of the corresponding genomic sequences in both 235th and 300th Asp residues of *sgg*-RD/RP/RQ isoforms were conducted in *w*^*1118*^ flies. Two single guide RNAs (sgRNAs) were used to introduce double strand breaks near by the edited genomic region and followed by homology-directed repair (HDR). The HDR donor plasmid was designed to harbor a DNA cassette containing the upstream homology arm of *sgg*, 3XP3-ScarlessDsRed flanking with PiggyBac terminal repeats, and the downstream homology arm of *sgg* with D235G/D300G mutations, which was constructed into the pUC57-Kan vector. The sgRNA and HDR donor plasmids used for microinjection were purified using the Plasmid Midi-prep kit (Qiagene). After validation of the CRISPR-knockin *sgg* alleles by genomic PCR coupled with Sanger sequencing, the ScarlessDsRed selection marker was then excised by PiggyBac transposon. The genomic PCR coupled with Sanger sequencing was performed to confirm the precise excision of ScarlessDsRed.

## Results

### Ex is required to suppress extra macrochaete in the scutellum

**Fig. 1:**
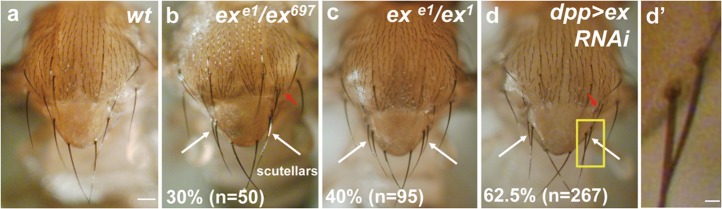
*ex* mutations promote generation of extra bristles in *Drosophila* notum. **a** Wild-type notal and scutellar bristle pattern. Ectopic bristles develop on the notum of transheterozygous combination of *ex* mutant alleles (**b**, **c**), and *ex RNAi* driven by *dpp-GAL4* flies (**d**). Note ectopic scutellar (white arrow) and post-alars (red arrow) bristles are observed. **d′** The enlargement of the boxed area in (**d**). The numbers indicate the percentages of the population of flies that contained extra macrochaetae in the scutellum. The allelic combinations of *ex* are used because they are hypomorphic mutants and can survive to adult with minimal growth defects. Scale bars for **a**–**d** 100 µm; **d′** 10 µm

**Fig. 2:**
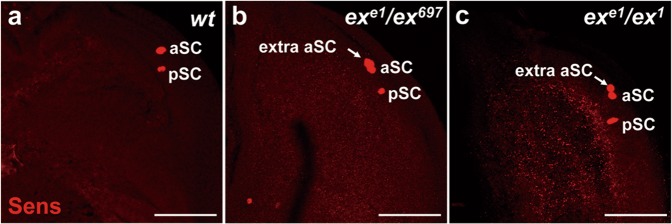
*ex* negatively regulates SOP cell formation. **a**–**c** The SOP cells in the scutellum of the third-instar larval wing disc are marked by Senseless (Sens, red, **a**–**c**). Note that ectopic anterior scutellar (aSC) SOP cells are visualized in allelic combination of *ex* mutants (**b**, **c**). Scale bars, 50 µm

Reduced *ex* function in *Drosophila* by using transheterozygous *ex* mutants caused the appearance of ectopic macrochaete on the notum (Fig. 1b, c). Knockdown of *ex* in the scutellum, using the *dpp-GAL4* driver, also resulted in the formation of extra macrochaete in 62.5% of flies (Fig. 1d). Compared with normal macrochaete, the extra macrochaete observed in *ex* mutants were occasionally thinner and shorter, but still contained socket cells of normal morphology (Fig. 1d’). These hypomorphic *ex* genotypes survived to adulthood without obvious growth defects in the scutellum (Supplementary Fig. 1). To address whether the extra macrochaetae were produced from extra SOP cells, the SOP cells were visualized by Senseless (Sens) staining. Normally, two sets of SOPs (one anterior scutellar (aSC) and one posterior scutellar (pSC) bristles, respectively) exist on the scutellum of one wing imaginal disc, whereas more than two SOP cells were detected when *ex* was downregulated (Fig. 2). These results indicate the extra macrochaetae of *ex* mutants are derived from extra SOP cells, not caused by a defect in bristle differentiation or SOP asymmetric division.

### Extra macrochaete formation requires Diap1 activity in an ex-dependent manner

**Fig. 3:**
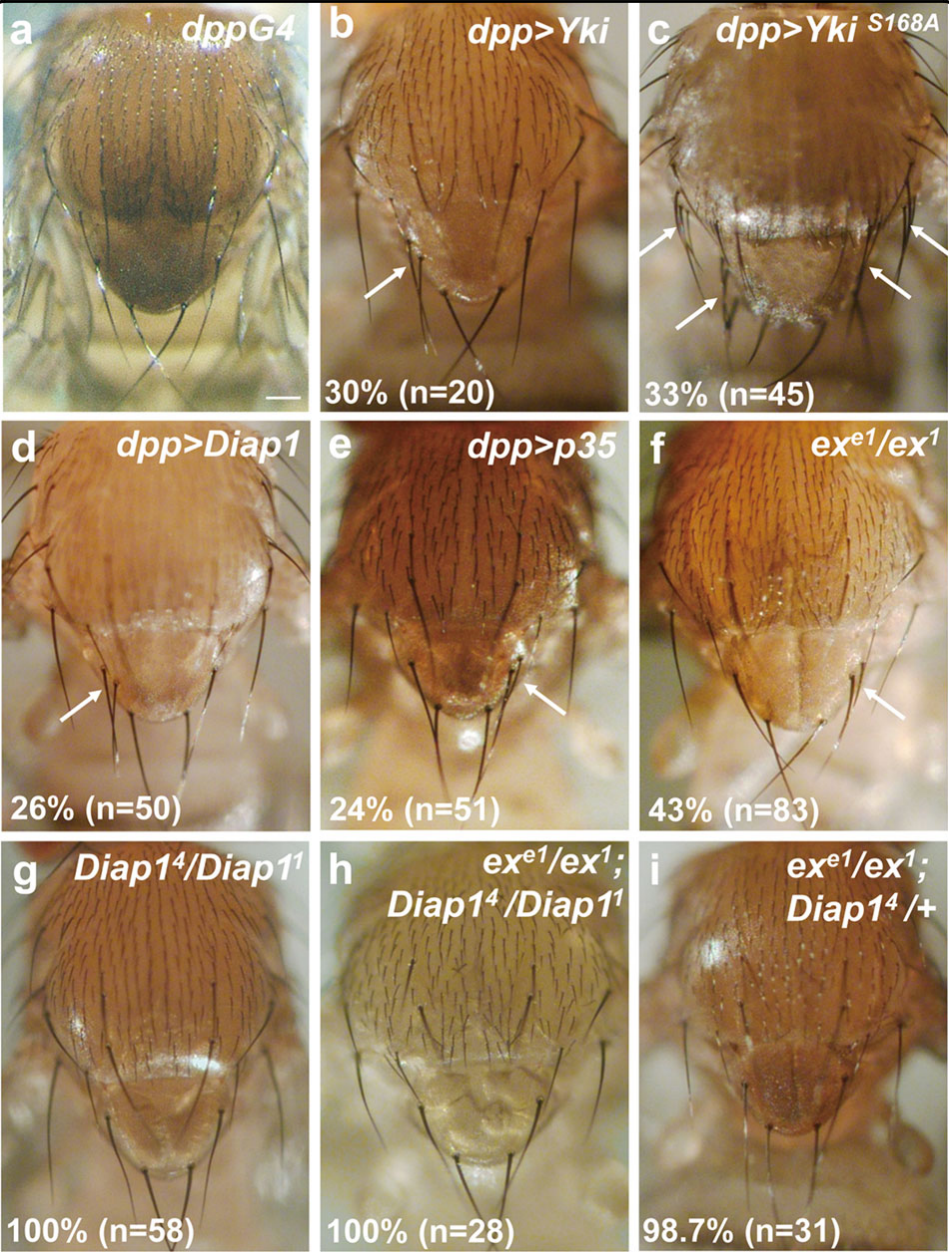
Downstream effectors of Hippo pathway mediate extra bristle formation. **a** Adult notum of *dpp-GAL4*. Overexpression of Yki (**b**), Yki^S168A^ (constitutive active Yki, **c**), Diap1 (**d**), p35 (**e**) induces extra macrochaetae. **f**, **g** Bristles patterning on the notum of transheterozygous combination of *ex* or *Diap1* mutant alleles. **h**, **i** Bristle patterning on the notum of double mutant combination of *ex* and *Diap1* alleles. Note that ectopic scutellar bristles on notum of *ex* mutant combination are restored when *Diap1* levels are decreased. The numbers in **a**–**f** indicate the percentages of the population of flies that contained extra macrochaetae in the scutellum, and arrow indicates an extra macrochaetae. The numbers in **g**–**i** indicate the percentages of the population of flies that have normal bristle patterning. Scale bar, 100 µm Figure 3a is a wrong image. I have included an updated Figure 3 (Fig 3_Sept07) with the correct Figure 3a in the attachment (and also sent you this updated file by email). Please make correction with the updated Figure 3.

**Fig. 4:**
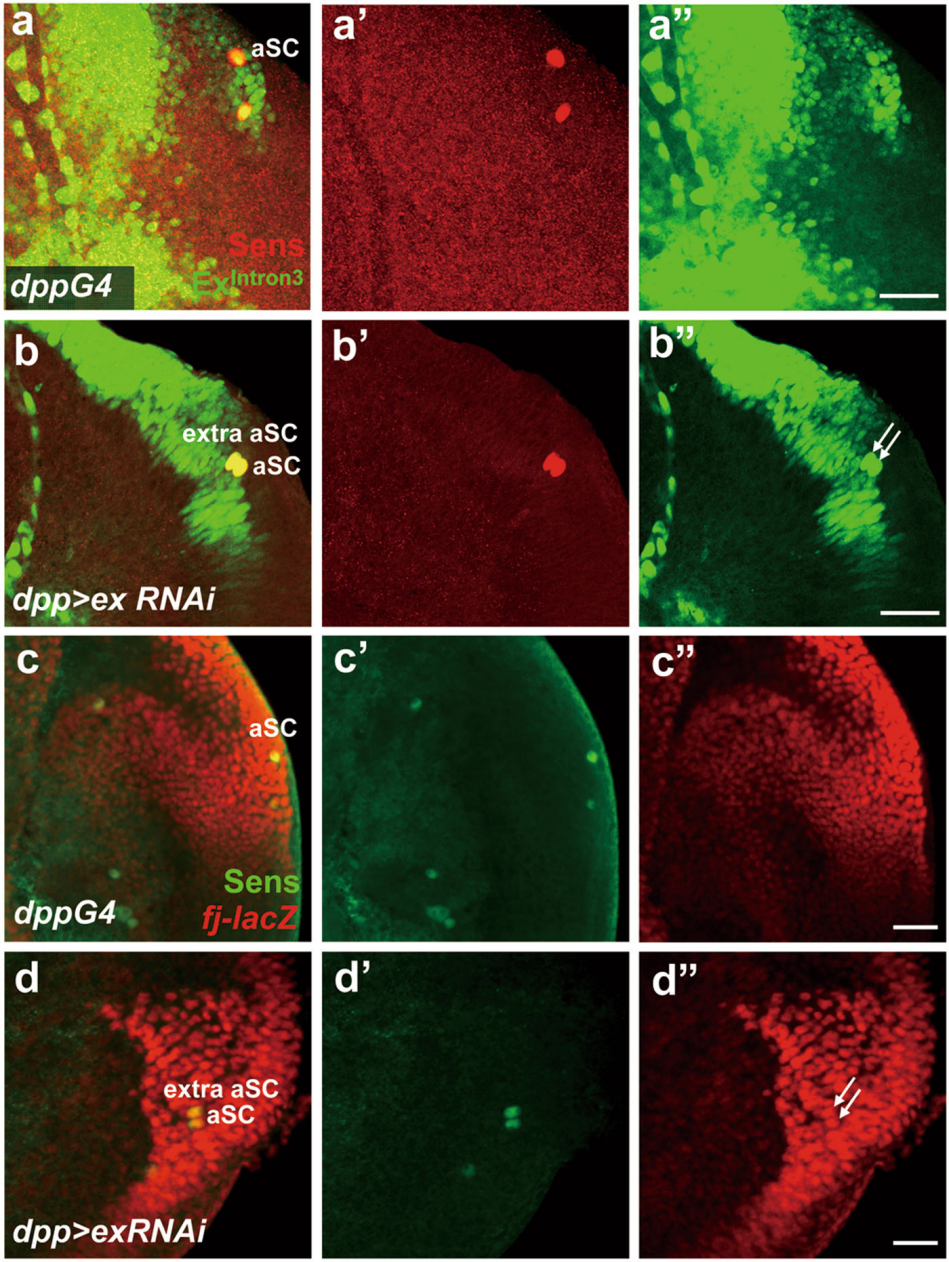
SWH activity is correlated with ectopic SOP cell. The SOP cells in the scutellum of the third-instar larval wing disc are marked by Sens (red) and Ex^Intron3^-GFP (green) in *dpp-GAL4* (**a**), and *dpp* *>* *ex RNAi* flies (**b**). The SOP cells in the scutellum of the third-instar larval wing disc are marked by Sens (green) and *fj-lacZ* (red) in *dpp-GAL4* (**c**), and *dpp* *>* *ex RNAi* flies (**d**). White arrow indicates aSC SOP cells (**d**). Scale bars, 20 µm

Since Ex functions upstream of the core kinase cassette to regulate SWH pathway activation^17^, mutations of *ex* led to inhibition of SWH signaling pathway and activation of Yki activity. Consistent with loss-of-function phenotype of *ex*, ectopic macrochaetae often appeared on the scutellum of flies overexpressing wild-type Yki or the activated Yki^S168A^ (Fig. 3b, c and Supplementary Fig. 2). These observations suggest that Yki activity is sufficient to induce extra bristle formation on scutellum. Moreover, overexpression of a Yki target gene, *Diap1*, caused extra scutellar bristles under the control of different GAL4 drivers (Fig. 3d, Supplementary Fig. 2 and^23^). These data suggest that the SWH pathway is involved in *ex*-dependent bristle inhibition through modulating *Diap1* activity. SWH activity was monitored by using *ex* intron 3 enhancer (referred to *Ex*^*Intron*^*-GFP*) reporter^18,19^, and also *fj-lacZ*, both of which report Yki activity. It was difficult to see overall changes in the notum region, although both reporters were present in the ectopic aSC SOP cell (Fig. 4). Collectively, our findings indicate that Yki activation contributes to ectopic macrochaetae formation.

To determine the involvement of *Diap1* in the *ex* mutant phenotype, *Diap1* levels were manipulated in *ex* mutants. As expected, lowered *Diap1* levels rescued the extra bristle phenotype of *ex* mutants while *Diap1* transheterozygous mutants have normal patterning and bristle numbers on the notum (Fig. 3f–i). These data indicate that *Diap1* is required for extra bristle formation in the absence of *ex*. The well-characterized function of *Diap1* is its role as a caspase inhibitor. To determine the role of caspase activity during bristle determination, caspase activity was blocked by expressing the antiapoptotic proteins baculovirus p35 or dominant-negative Dronc under the control of *dpp-GAL4* (Fig. 3e and data not shown). Indeed, blockage of caspase activity led to ectopic bristle formation, which is consistent with previous reports^23^. Since many studies have shown that caspase activation negatively regulates macrochaetae development^23,31–33^, the involvement of *Diap1* in bristle formation is likely to be through inhibition of caspase activity. Such caspase-dependent macrochaetae regulation has been shown to represent an apoptosis-independent process^23,34^. Like previous authors, we also found no evidence for apoptosis in normal SOP patterning (data not shown).

### Wg signaling modulates *ex*-dependent bristle phenotypes

**Fig. 5:**
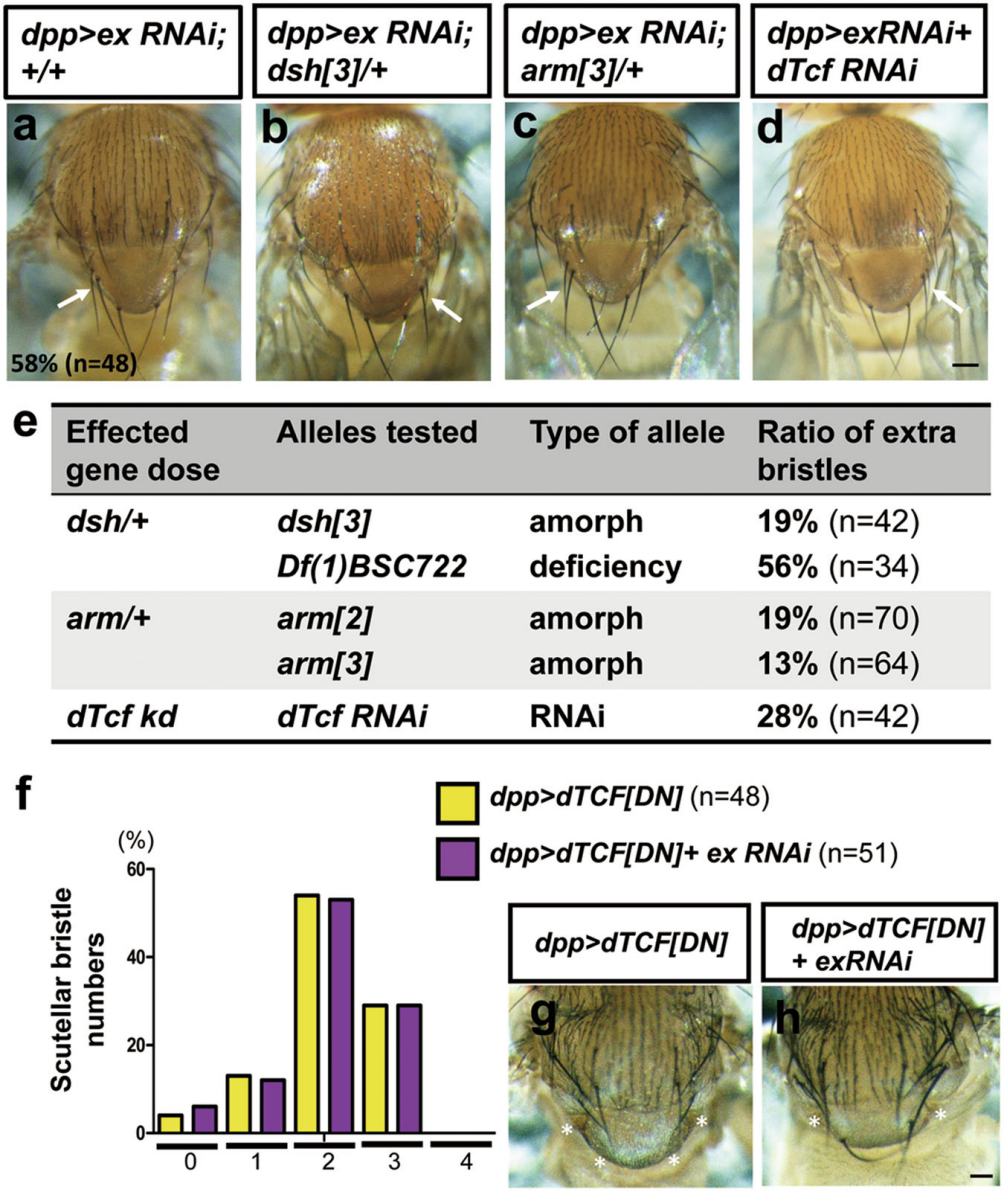
Genetic interaction between *ex* and the Wg pathway mutations. **a** Adult notum of *dpp-GAL4, UAS-ex-RNAi*. **b**–**d** Extra scutellar bristles caused by knockdown of *ex* are suppressed in decreased levels of Wg pathway components. Genotypes: *dsh*^*3*^*/+;dpp-GAL4,UAS-ex-RNAi/+*; *arm*^*3*^*/+;dpp-GAL4,UAS-ex-RNAi/+*; *w;dpp-GAL4,UAS-ex-RNAi/UAS-dTcf-RNAi*. **e** A table summarized data of **b**–**d** and *Df(1)BSC722/+;dpp-GAL4,UAS-ex-RNAi/+*; *arm*^*2*^*/+;dpp-GAL4,UAS-ex-RNAi/+*. The numbers indicate the percentages of the population of flies that contained extra macrochaetae. **f** *X* axis represents scutellar bristle numbers 0–4. *Y* axis represents the penetrance of indicated scutellar bristle numbers. Wild-type flies normally have four scutellar bristles while overexpression of *dTCF[DN]* results in a loss of scutellar macrochaetae. Adult nota of *w;UAS-dTCF[DN]/+;dpp-GAL4/+* (**g**) and *w;UAS-dTCF[DN]/+;dpp-GAL4,UAS-ex-RNAi/+* (**h**). Loss of scutellar macrochaetae is indicated by asterisk. Scale bars, 100 µm

SOP cells of the macrochaetes arise from proneural clusters in the wing imaginal disc during larval stage. Spatial and temporal patterning of the proneural clusters are established by the expression of proneural genes *achaete* and *scute*, which is controlled by multiple *cis*-regulatory elements distributed throughout the *achaete* and *scute* transcription units to permit the precision of SOP cell specification^35–37^. Among the critical regulators in modulating proneural gene expression, previous studies have identified Wg/Wnt signaling in particular as affected by nonapoptotic caspase activity during thoracic bristle patterning^23^. Sgg/GSK3β is a negative regulator of Wg/Wnt signal transduction. *ex* and *sgg* mutations shared similar phenotypes in bristle formation (this study;^23,38^). Independent evidence also points out that Wg signaling is altered in the absence of *ex* in eye discs^39^. Genetic analyses were performed to verify the involvement of Wg signaling cascade in *ex*-mediated SOP specification. Indeed, the extra bristle phenotype caused by downregulating *ex* was suppressed by removing one copy of Wg signaling components (Fig. 5a–c, e). Similar results were also found when dTcf was knocked down in *dpp* *>* *ex RNAi* flies (Fig. 5d). Overexpression of dominant-negative dTCF (dTCF[DN]) alone prevented macrochaetae formation (yellow bar in Fig. 5f–h). The penetrance of scutellar bristle loss was comparable in *ex*-knockdown flies with overexpressing dTCF[DN] transgene (magenta bar in Fig. 5f). Although the Notch pathway has been shown to play important role in bristle development^40^, E(spl) expression was not changed when ex was mutated (Supplementary Fig. 3), consistent with previous conclusions that *ex* mutations do not reduce Notch signaling^41^. These results suggest that Wg signaling pathway acts downstream of *ex* in SOP cell formation, and therefore that the SWH pathway may by the source of the nonapoptotic caspase activity that acts on Wg signaling.

**Fig. 6:**
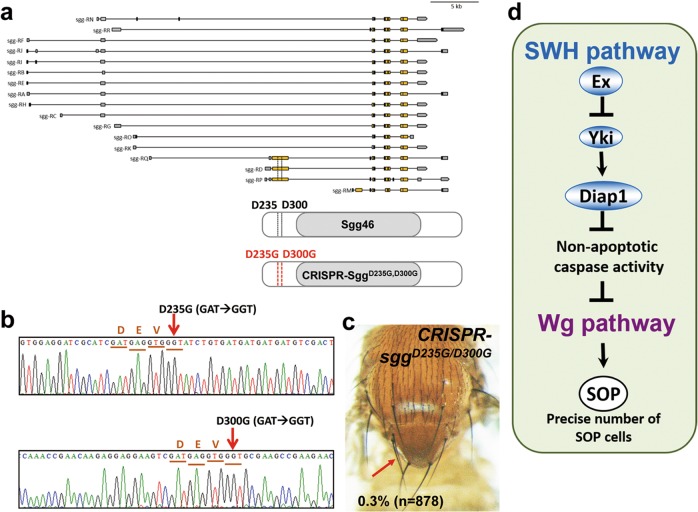
Sgg is not the only target of nonapoptotic caspase activity. **a** Schematic representation of the D235 and D300 locus in 3 of 17 sgg isoforms. Exons are represented as yellow boxes, and introns are represented by lines. Noncoding regions are shown as gray boxes. **b** Sanger sequencing of *CRISPR-sgg*^*D235G/D300G*^ genomic DNA. Note that the corresponding sequences of DEVD235th and DEVD300th residues are mutated from GAT to GGT (glycine). **c** Bristle patterning of *CRISPR-sgg*^*D235G/D300G*^ flies. Note the ectopic scutellar bristles (indicated by red arrow) are found in a very low penetrance. **d** Model of integrated pathways in regulating SOP specification. Components involving in signal transduction of SWH and Wg pathways are indicated in blue and purple, respectively. The crosstalk between these two pathways is nonapoptotic caspase activity

A caspase-dependent cleavage resulting from caspase activation mediates nonapoptotic signaling in determining SOP cells. This caspase-dependent cleavage is thought to activate Sgg/GSK3β during SOP cell formation^23^. Previous studies have reported that caspase-dependent cleavage occurs at the DEVD motif, which has been mapped to DEVD^235^ and DEVD^300^ of Sgg (Sgg46 isoform) protein^23,42^. Hence, we hypothesize that the caspase inhibitor Diap1 might be the critical effector connecting SWH pathway with Wg pathway through modulating kinase activity of Sgg to determine the correct number of SOP cells. To probe the involvement of Sgg in this regulation in depth, we generated an in vivo noncleavable form of Sgg (*sgg*^*D235G/D300G*^) using CRISPR-Cas9 technique (thereafter referred to as *CRISPR-sgg*^*D235G/D300G*^, Fig. 6a). The sequence validation of *CRISPR-sgg*^*D235G/D300G*^ was performed in both genomic DNA and cDNA (Fig. 6b and Supplementary Fig. 4a). If caspase cleavage of DEVD^235^ or DEVD^300^ was required to activate Sgg and inhibit Wg signaling during normal bristle development, then *sgg*^*D235G/D300G*^ flies should have elevated Wg signaling and extra bristles, but extra macrochaetae were observed at only a low frequency and only in the first few generations. In case there might be selection for genetic modifiers suppressing the phenotype, we selected *sgg*^*D235G/D300G*^ flies where proximal parts of the X chromosome were replaced with those from the FRT19 strain after meiotic recombination, and completely exchanged the autosomes by outcrossing at every generation. These flies, which should only be able to retain genetic modifiers on one section of the X chromosome, exhibited only three extra thoracic macrochaetae in 878 hemizygous *sgg*^*D235G/D300G*^ males (Fig. 6c), a much lower frequency than observed when caspase activity was inhibited (Fig. 3d). Penetrance was similarly low in transheterozygous *sgg*^*1*^*/CRISPR-sgg*^*D235G/D300G*^ females (Supplementary Fig. 4b). When *ex* was knocked down in the *CRISPR-sgg*^*D235G/D300G*^ background, the frequency of extra scutellar macrochaetae was not affected by the *sgg* mutant background (Supplementary Fig. 4c, d). These observations suggest that Sgg is not the major substrate of caspase-dependent cleavage that affects numbers of macrochaetae in the scutellum. Intriguingly, overexpression of *UAS-sgg*^*D235G/D300G*^ using *Sca-Gal4* resulted in ectopic macrochaete in 19.3% of flies^23^. This much higher frequency than observed in *CRISPR-sgg*^*D235G/D300G*^ flies suggests that overexpression of *UAS-sgg*^*D235G/D300G*^ might have dominant negative effects. In sum, our data indicate that there is another target of caspases that affects Wg signaling. Our data cannot rule out some contribution of Sgg cleavage that is redundant with the other target(s).

## Discussion

Unlike the well-known concept that the key roles for SWH pathway are in the regulation of cell proliferation and organ size, the present study reveals a novel function of SWH signaling in cell fate determination through nonapoptotic caspase signaling. As is well-known, deregulation of SWH pathway leads to the activation of the Yki target gene, *Diap1*, which restrains caspase activity. Although this can regulate cell survival, here we report that SWH and Yki play a role in normal development in suppressing nonapoptotic caspase activity. In the *Drosophila* thorax, nonapoptotic caspase activity is needed to suppress activity of Wg signaling and restrain SOP cell specification^23^. This is here shown to depend on the SWH pathway, without which Diap1 expression is too high to permit normal patterning. While this paper was under review, another study reported that nonapoptotic caspase activity is also regulated by the SWH pathway, during tracheal development^43^.

During SOP specification, caspase activity is transiently controlled by the turnover of Diap1. Diap1 degradation is triggered by the *Drosophila* IKK-related kinase (DmIKKε)-dependent phosphorylation. In consistent with phenotype of high levels of Diap1, downregulation of DmIKKε led to extra macrochaetae formation^44^. Hence, the levels of Diap1 play determinant role in cell fate specification. Here we provide evidence that transcriptional regulation of *Diap1* by Yki and the SWH pathway is also important for SOP cell determination, which is disrupted by hypomorphic mutations of *ex*, which affect the level of Yki activity.

The *Drosophila* GSK3β ortholog, Sgg, was identified as a potential substrate for caspase-dependent cleavage. One isoform, Sgg46, is inactive but can be cleaved into the active isoform Sgg10, which negatively regulates Wg signaling through the phosphorylation and degradation of Arm^23^, and also directly phosphorylate Scute and its activator Pannier in SOP cell specification^45^. Caspases potentially have hundreds of substrates, but Sgg46 was believed to be significant for Wg signaling and SOP patterning because overexpression of a form with mutated caspase sites, SggD235G/D300G, phenocopied blockade of nonapoptotic caspases by p35 overexpression^23^. In fact, SggD235G/D300G overexpression was quantitatively less effective than completely blocking caspases with p35, but this was attributed to the simultaneous presence of wild-type Sgg46 encoded by the endogenous locus^23^. It was presumed that the overexpressed, SggD235G/D300G protein behaved as a competitive inhibitor of Sgg46 cleavage. If this model was correct, we would expect that modifying the endogenous *sgg* locus to encode Sgg^D235G/D300G^ (which would not affect other, shorter isoforms of Sgg, Fig. 6a), should more completely prevent cleavage of Sgg46 and more completely block the nonapoptotic caspase regulation of Wg signaling and SOP patterning, resulting in many extra macrochaetae, comparable with ectopic expression of Diap1 or p35. In contrast to this expectation, we found almost no phenotypic effect of the endogenous *sgg*^*D235G/D300G*^ mutant. This is not consistent with the model that Sgg46 is the main target of nonapoptotic caspase signaling in bristle patterning. Although we cannot exclude that Sgg46 might be activated by cleavage at another site, this would not explain why Sgg46 could not be activated when SggD235G/D300G protein was overexpressed. Therefore, we conclude that nonapoptotic caspases regulate one or more other substrates that are critical for Wg signaling and SOP patterning, and that SggD235G/D300G overexpression is a competitive inhibitor of cleavage of these other substrates.

A recent study has reported that the unconventional myosin Crinkled acts as an adapter to facilitate Sgg46 cleavage and activation by Dronc^46^. Aside from our finding that the caspase cleavage sites of Sgg46 are largely dispensable for bristle patterning, the model that Sgg46 is a Dronc target also does not fit with the observation that bristle patterning is disrupted by p35 overexpression^23^, since p35 does not inhibit Dronc. The ectopic p35 phenotype strongly suggests that the major regulators of bristle patterning are substrates of p35-dependent effector caspases, not direct Dronc targets. These data could explain how SggD235G/D300G overexpression is dominant negative, however, if SggD235G/D300G inhibits Dronc and Crinkled function, leading to deficient nonapoptotic signaling by downstream effector caspases.

Our main conclusion is that, in addition to its previously known roles, the SWH pathway is important for regulating nonapoptotic caspase signaling, presumably through Yki control of *Diap1* transcription. In the *Drosophila* thorax this is required to restrain Wg signaling and bristle patterning (Fig. 6d). It is possible that this nonapoptotic caspase signaling might underlie the crosstalk between SWH and Wg signaling in other tissues, such as the eye and wing, the molecular basis of which has so far remained unclear. At one time it was thought that *wg* was a transcriptional target of Yki but it is now thought this reflects enhanced *wg* autoregulation when Wg signaling is elevated^39,47^. Crosstalk between SWH and Wnt signaling appears to be conserved, having also been reported in vertebrates^48–54^. The mechanisms that have been suggested in vertebrates are not yet known to involve nonapoptotic caspase signaling, however.

It is striking that in both *Drosophila* and in mammals, SWH signaling and nonapoptotic caspase signaling both are implicated in neuronal morphogenesis. SWH signaling affects synapse development and dendrite morphogenesis^55–59^, while nonapoptotic roles of caspases remodel *Drosophila* dendritic arborization neurons and regulate axon degeneration in mammals. Defects of caspase-dependent nonapoptotic signaling affect plasticity and result in disease such as Alzheimer’s disease^60,61^. Wnt signaling is also involved in neuronal development and has been associated with neurological diseases including Alzheimer’s disease, Parkinson’s disease, schizophrenia, and autism^62–66^. Crosstalk between SWH signaling, Wnt signaling and caspase-dependent nonapoptotic signaling may contribute to the molecular mechanisms of neuronal pathogenesis. This crosstalk may occur in multiple processes during development, in light of the finding that DrICE has a nonapoptotic function, which acts downstream of the SWH signaling to regulate endocytic trafficking during tracheal morphogenesis^43^.

## Supplementary information

Extra scutellar bristle is not caused by growth defect in the depletion of ex

Inactivation of caspase or Hippo pathway is involved in extra bristle formation

Expression of Notch target is not changed in ex mutant

Sgg is not the only target of non-apoptotic caspase activity
