# Correction: Salvador–Warts–Hippo pathway regulates sensory organ development via caspase-dependent nonapoptotic signaling

**DOI:** 10.1038/s41419-019-2029-8

**Published:** 2019-10-22

**Authors:** Lan-Hsin Wang, Nicholas E. Baker

**Affiliations:** 1 Graduate Institute of Life Sciences, National Defense Medical Center, 161 Sec 6, Minquan E. Rd, Taipei, 11490 Taiwan; 2 Department of Genetics, Albert Einstein College of Medicine, 1300 Morris Park Avenue, Bronx, NY 10461 USA; 3 Department of Developmental and Molecular Biology, Albert Einstein College of Medicine, 1300 Morris Park Avenue, Bronx, NY 10461 USA; 4 Department of Ophthalmology and Visual Sciences, Albert Einstein College of Medicine, 1300 Morris Park Avenue, Bronx, NY 10461 USA

**Keywords:** HIPPO signalling, Differentiation

**Correction to: Cell Death & Disease**

10.1038/s41419-019-1924-3, published online 11 September 2019

Following publication of this article, it was brought to our attention that there was a typo in the References (reference number 43) whereby the first author’s name was misspelled. The correct citation is provided below. We apologize for the inconvenience.

43. McSharry, S.S. & Beitel, G.J. The Caspase-3 homolog DrICE regulates endocytic trafficking during *Drosophila* tracheal morphogenesis. *Nat Commun*. 10, 1031 (2019)

This has been corrected in both the PDF and HTML versions of the Article.

