# Supplementary figures and images for: Salvador–Warts–Hippo pathway regulates sensory organ development via caspase-dependent nonapoptotic signaling
Source: Cell Death Dis. 2019 Sep 11;10(9):669. doi: 10.1038/s41419-019-1924-3 (PMC6739336; doi:10.1038/s41419-019-1924-3)

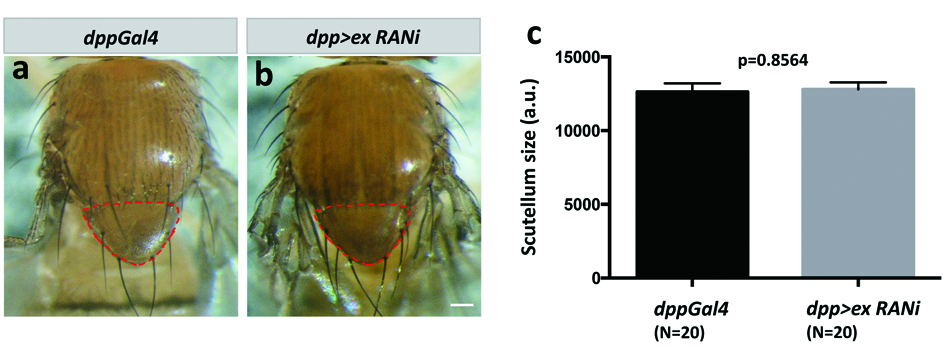

Supplement: Supplementary file 1 — Extra scutellar bristle is not caused by growth defect in the depletion of ex [file 41419_2019_1924_MOESM1_ESM.tif]

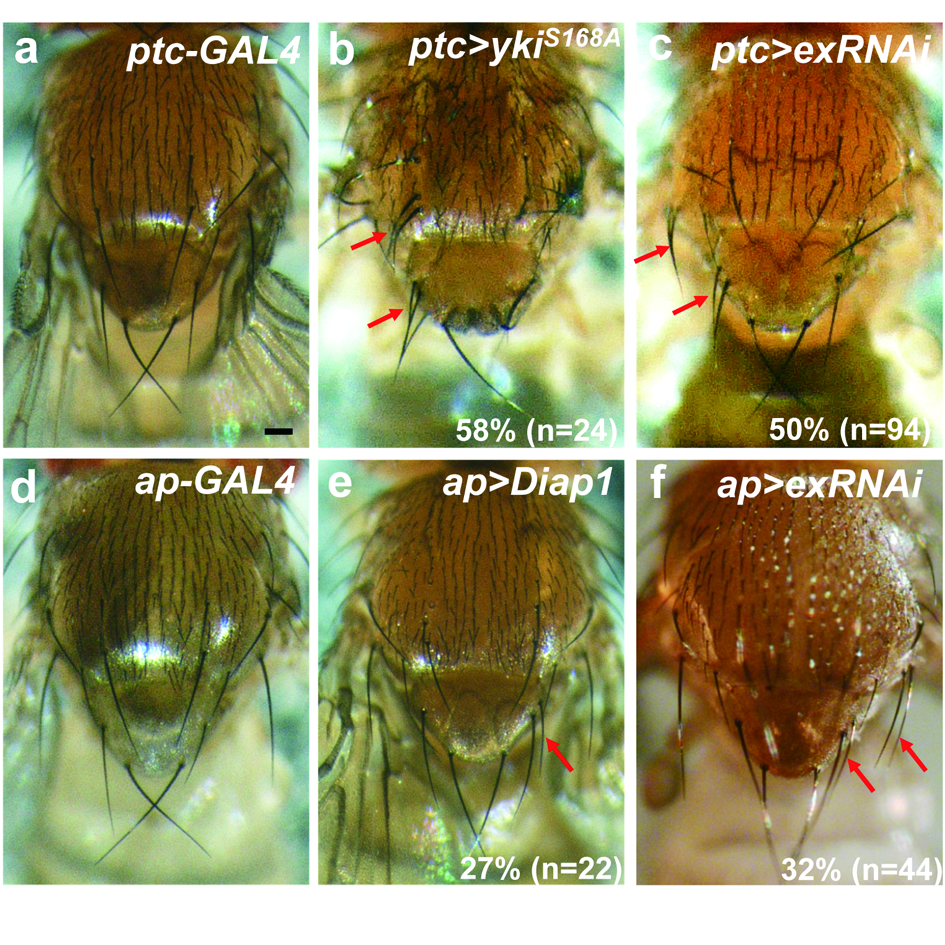

Supplement: Supplementary file 2 — Inactivation of caspase or Hippo pathway is involved in extra bristle formation [file 41419_2019_1924_MOESM2_ESM.tif]

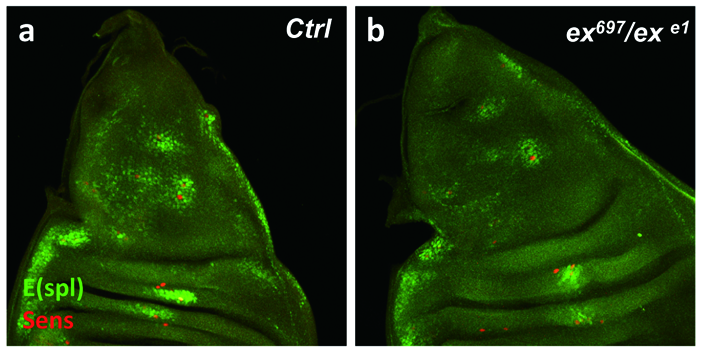

Supplement: Supplementary file 3 — Expression of Notch target is not changed in ex mutant [file 41419_2019_1924_MOESM3_ESM.tif]

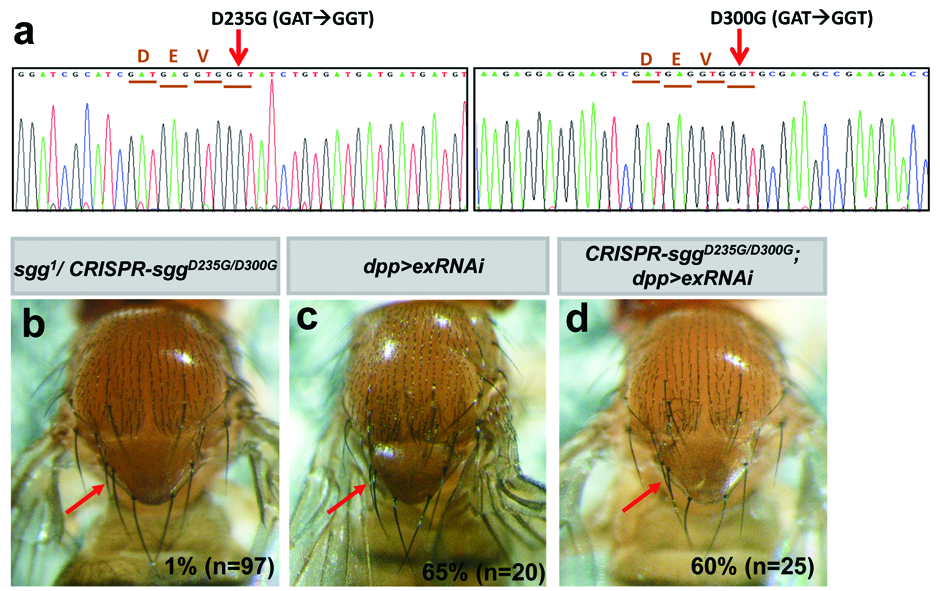

Supplement: Supplementary file 4 — Sgg is not the only target of non-apoptotic caspase activity [file 41419_2019_1924_MOESM4_ESM.tif]
